# Computer-assisted medical history taking prior to patient consultation in the outpatient care setting: a prospective pilot project

**DOI:** 10.1186/s12913-024-12043-3

**Published:** 2024-12-18

**Authors:** Roman Hauber, Maximilian Schirm, Mirco Lukas, Clemens Reitelbach, Jonas Brenig, Margret Breunig, Susanne Brenner, Stefan Störk, Frank Puppe

**Affiliations:** 1 Department Clinical Research & Epidemiology, Comprehensive Heart Failure Center, University Hospital Würzburg, Würzburg, Germany; 2 Department Internal Medicine I, University Hospital Würzburg, Würzburg, Germany; 3 Würzburg, Germany; 4 Human Computer Interaction, Faculty of Media, Bauhaus University, Weimar, Germany; 5 Computer Vision Laboratory, Center for Artificial Intelligence and Data Science, University of Würzburg, Würzburg, Germany; 6 Joint Center for Nephrology & Cardiology, Wertheim/Tauberbischofsheim, Germany; 7 Federal Police Academy, Oerlenbach, Germany; 8 Chair for Artificial Intelligence and Knowledge Systems, University of Würzburg, Würzburg, Germany

**Keywords:** Digital health, Clinical documentation, Knowledge-based system, Technological anamnesis concept, Interoperability, Pilot project, Feasibility, Physician–patient relationship, Research data infrastructure

## Abstract

**Background:**

Feeding patients’ self-reported medical history into the diagnostic care process may accelerate workflows in clinical routine.

**Methods:**

We prospectively piloted a novel medical history documentation system in a German cardiological outpatient practice and evaluated its feasibility and perceived usefulness. Based on a generic software that allows to record structured information, a customized solution for the cooperating practice was developed and implemented. Prior to the consultation of the physician, the patient used a tablet that guided the user through a structured comprehensive workflow to document the medical history. The retrieved information was arranged by the software into a ready-to-use text format, presented to the physician in an editable form and added to her report. Three user-centered endpoints were explored: i) Appropriateness—measured by the duration of a patient interview; ii) Patient acceptance—assessed by three questions to patients; iii) Usefulness—operationalized by multiple ratings of the physician.

**Results:**

A total of 2,513 patients were approached of which 2,415 provided complete histories. The system was assessed as appropriate for the practical workflow in terms of time and workflows. The patient-system interaction was rated favourably by patients including elderly ones. The system was regarded useful by the physician, reducing her daily workload by about one hour.

**Conclusions:**

Automated history-taking tools deployed before consultation could support physicians in obtaining patients’ medical histories, thereby reducing professionals’ perceived workload. The technical and methodological limitations of our study should be respected, calling for additional future evaluations.

**Supplementary Information:**

The online version contains supplementary material available at 10.1186/s12913-024-12043-3.

## Background

Taking a patient’s medical history marks the typical starting point of a patient-physician contact as it provides pivotal information for optimizing the diagnostic and treatment approach. The time that can be allotted for the taking of a medical history frequently is a clinical-ethical and logistical challenge, reflecting the delicate equilibrium between the quality and efficiency of medical practice [1–4].

One concept to advance the efficiency of medical care delivery is to relieve physicians of as many tasks as possible that do not require a physician´s knowledge and/or attention. In this regard, the digitalization of medicine holds promise to simplify such processes, but medical care in Germany is still poorly digitalised. In 2020, 69% of German hospitals exhibited a score value of zero on the “Electronic Medical Records Adoption Model” (EMRAM; score range: 0 – not digitalised to 7 – fully digitalised), which assesses the digitalisation of hospitals [5]. This is an important barrier, as the standardised documentation of planned and performed tasks constitutes the application area, which is likely to benefit the most from the advances in digitalisation. Indeed, documentation has become one of the most time-consuming factors in the clinical routine of care providers [6, 7].

When it comes to the practical implementation of changes to IT processes or digital services, many stakeholders, including medical practices, appear to have reservations as they encounter and/or expect shortcomings [8, 9]. By contrast, a market-oriented survey in Germany showed that physicians in private practice appear to have a positive attitude towards digital tools, in general [10]. This survey also emphasised that newly implemented structures and applications have to meet certain demands for user acceptance, including a reduced workload, perceived user benefit, and a firm integration into clinical routine. Furthermore, if such applications involve patient interaction, they need to be convinced of the innovation, too. On the other hand, for a successful and sustainable implementation of IT supported processes a medical practice, the users have to have realistic expectations. Studies conducted in German primary care revealed that increased interoperability, continued technical support and improved usability are desired, whereas workflow adjustments, reimbursement issues, and the need for extensive training constitute major barriers when it comes to the implementation of digital health solutions [11]. Moreover, there are intrinsic personal attributes, such as the digital proficiency of personnel, extroversion, or the anticipated advantages of a digital tool, which drive the digital evolution and maturation of a practice [12]. In view of these results, close monitoring of the implementation and early reaction to potential pitfalls of a digital solution is important.

The here presented project piloted the implementation of a novel medical history taking system in a German cardiological outpatient practice that interacted with the patient prior to consultation with the physician. The system used a dynamic questionnaire and a semi-automated text generation module to accelerate and simplify medical documentation. Adopting a user-centred approach, we aimed to evaluate its appropriateness, perceived usefulness, and patient acceptance.

## Methods

The current project piloted an innovative software solution, in the following called “INTERVIEW system”, in a routine medical setting. The generic software package was customised to the specific needs of the cooperating cardiology practice to support the structured retrieval and documentation of diverse types of information. For the here reported use case, the INTERVIEW system was customised to serve two main purposes: a) to comprehensively and efficiently record the self-reported history of outpatients prior to the face-to-face visit taking place with the consulting physician, and b) to then transfer the retrieved information into a ready-to-use text format for documentation in the physician report.

### Project design and approach to data collection

#### Main objectives

The overarching goal of this project was to customise the generic software to a) allow obtaining and storing standardised and harmonised self-reported anamnestic information from patients, b) redact, and transform this data into meaningful text answers that can directly become part of the physician´s report, and c) provide anonymised information prior for analysis that allows to assess the approach in a practical setting as perceived by patient and physician.

#### Setting and development team

This prospective, monocentric and observational data collection project was embedded into routine care at one cardiology practice located in a rural area in the vicinity of the city of Würzburg, Germany. The participating cardiology practice strived to optimise its patient care pathway and agreed to the cooperational project. The participating physician, a specialist trained in cardiology with over 15 years of clinical experience including research activities, owned the practice and was kept involved into all development and implementation decisions made both in the pre-test phase and pilot phase (entire project duration about two years). The development team also included a user experience expert, who evaluated the user interfaces regarding heuristics.

#### Project phases and patient selection

**Fig. 1:**
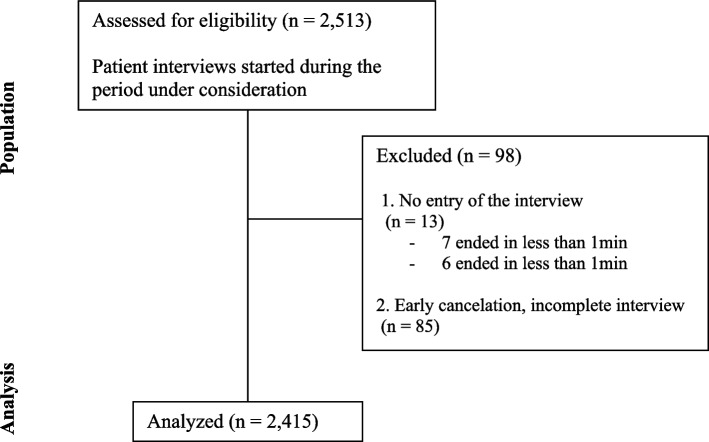
Flow for data inclusion and analysis

Since the system was intended to be used by persons of all age groups regardless of their technical affinity and because it was assumed that older people could encounter more and particular difficulties with the system, a pre-test phase was run to test the accessibility of the INTERVIEW system in elderly participants. This input triggered adaptions resulting in a more user-friendly and intuitive design. Thereafter, between July 2020 and August 2021, all patients entering the practice with a full appointment involving the taking of their history were considered eligible and asked to use the INTERVIEW system in order to interrogate their history electronically. All completed consultations during the supervised project period were included in the data analysis. The flow for the data inclusion is shown in Fig. 1. The following value ranges were defined in advance for the basic data of the descriptive statistics in order not to distort the results due to potential input mistakes: age [18; 110]; weight [40; 180]; height [120;220].

#### Clinical endpoints and their operationalisation

For the effective implementation of technical innovations in medicine, it is essential to earn the support of users. Three endpoints addressing three important domains informed on the feasibility of the process being deployed in clinical routine care: i) appropriateness, ii) patient acceptance, and iii) usefulness. The operationalisation of endpoints was as follows:

#### Domain “appropriateness”

The application must meet the requirements of clinical routine and comply with the time constraints posed by the procedures and processes of the respective practice. As a surrogate, the completion time of the surveys by the patient was measured (in minutes:seconds). The INTERVIEW system calculates the completion time as the difference between the start of a survey and the final saving by the medical assistant. The average waiting time for a patient at a cardiology practice before the first physician contact specialist was assumed at 30 min as consensus of the cardiologists of the project team. Therefore, provided that the interview remained within this time span, it is judged appropriate.

#### Domain “patient acceptance”

Three questions to evaluate the perception and acceptance were presented to all patients interviewed on the tablet after the content questions: 1. “How helpful do you feel the questions asked are in terms of assessing your complaints?” (Answers: “Very helpful” vs “Rather helpful” vs “Rather not helpful” vs “Not helpful”; 2. “Do you find a tablet survey more convenient than a paper survey?” (Answers: “Yes” vs “No” vs “I do not care); 3. “Do you think the survey prior to the physician visit has an advantage for my treatment?” (Answers: “Yes” vs “No”). If more than 80% of the cumulated answers within this domain are favourable the acceptance will be rated as given. 

#### Domain “usefulness”

Does the practice benefit from using the digital system, and, if yes, in which ways? At three points during the implementation period the physician was interviewed. The first two interviews were paper-based questionnaires (August 2020 and October 2020), that were to be completed when finalising the medical reports. The last interview was conducted as a conversation at the end of the implementation (August 2021). Three questions were asked. The physician was asked to grade, whether the texts contained errors (check boxes yes/no) and whether the texts were plausible (on a scale that used German school grades 1 to 6, i.e., from 1 = best to 6 = worst). The third item asked, whether she had been able to finish the work list with different speed compared to the period when the INTERVIEW system had not yet been in place (the following check boxes could be chosen: “significantly slower”, “slower”, “similarly fast”, “faster”, “significantly faster”). The questionnaires included a field for providing comments as free text. For the last, oral interview she was additionally asked whether she might give an estimate about the time saved according to the usage of the interview system. The evaluation form of the first two interviews and the script for the survey at the end can be found in the supplement (translated from German to English). If in the end plausibility and absence of errors are rated “very good” or “good” (i.e., grades 1 or 2) and a positive time saving is reported, the INTERVIEW system should be rated as useful. Provided that all three endpoints were met, the INTERVIEW system was regarded feasible.

### Components of the INTERVIEW system

**Fig. 2:**
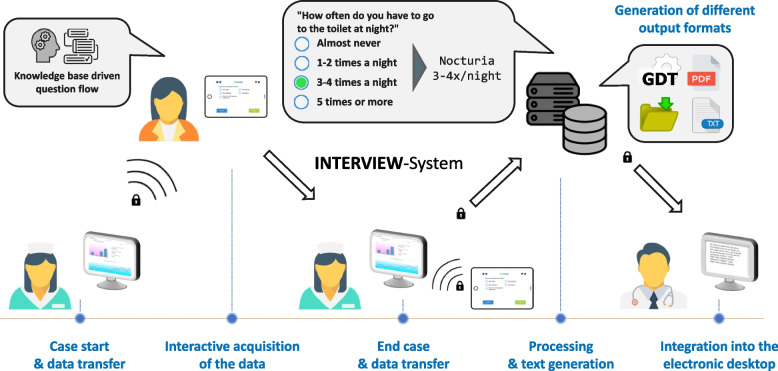
Schematic flow of the process of the INTERVIEW system deployment

The customised medical history taking system offers a technical solution for the collection and documentation of patient information in the context of the anamnesis. Using a tablet, patients are asked about their medical condition and previous illnesses. This information is then embedded in a flow text that serves as a suggestion for the documentation in the physician’s report. The INTERVIEW system consists of various components. In the following, “system” refers to the combination of tablet app used by the patients (“app”), a dashboard used by the staff, and server software (text generator, database etc.). The patient information captured on the tablet is processed by the software, and a continuous text block is then transmitted to the practice’s patient management system (PMS) via a standardised interface. Several tools with corresponding interfaces were used in the technical process. For a comprehensive overview, see Fig. 2.

#### Documentation pathway

The here applied electronically supported process is an evolution of an existing approach [13] and may be described as follows: When a new patient presents to the physician’s office, her/his record is reviewed by assistant staff as part of the regular operating procedure. On the patient record, a dedicated button enables starting the digital anamnesis session by sending the patient’s core information (name, ID) to the INTERVIEW system. Assistant staff selects the patient ID on a connected tablet and selects the questionnaire to be filled. The patient then receives the tablet displaying the first question and proceeds with answering all subsequently presented questions. Depending on the patient’s response of a particular question, additional adapted questions appear, whereas irrelevant questions are removed from the catalogue. The system is rule-based, i.e., it is determined in advance which content is to be queried for which combination of patient responses. Five types of questions were used: single choice, multiple choice, two numeric types, and a special panel question addressing “blood pressure”. Conceptually, it was decided that an asked question had to be answered by the patient in order to facilitate moving to the next question.^1^ When all questions have been answered, the patient is asked to return the tablet at the reception desk. Assistant staff then enters a password that allows to close, dismiss, or pause the case. The obtained set of answers is transformed into a continuous text, which the physician then can view, edit, and also use for documentation in the physician’s report. Additionally, the answers are added in an anonymised fashion to the data warehouse for later retrieval and quality control.

#### Interfaces

The continuous text and patient information are then transmitted via the device data transfer (German abbreviation: GDT) interface to the server on which the PMS is running. The GDT interface has been standardised for PMS and is supported by 84% of all PMS manufacturers in Germany [14]. GDT was integrated as a bidirectional interface, i.e., in addition to the transmission of the results into the digital patient record, it was also possible for the medical staff to request an anamnesis for a given patient directly from the PMS user interface and have the request directly appear on the connected mobile end device. The PMS stores the text in the respective patient file, so that it can be viewed and edited by the staff in a defined field*.*

#### Architecture

**Fig. 3:**
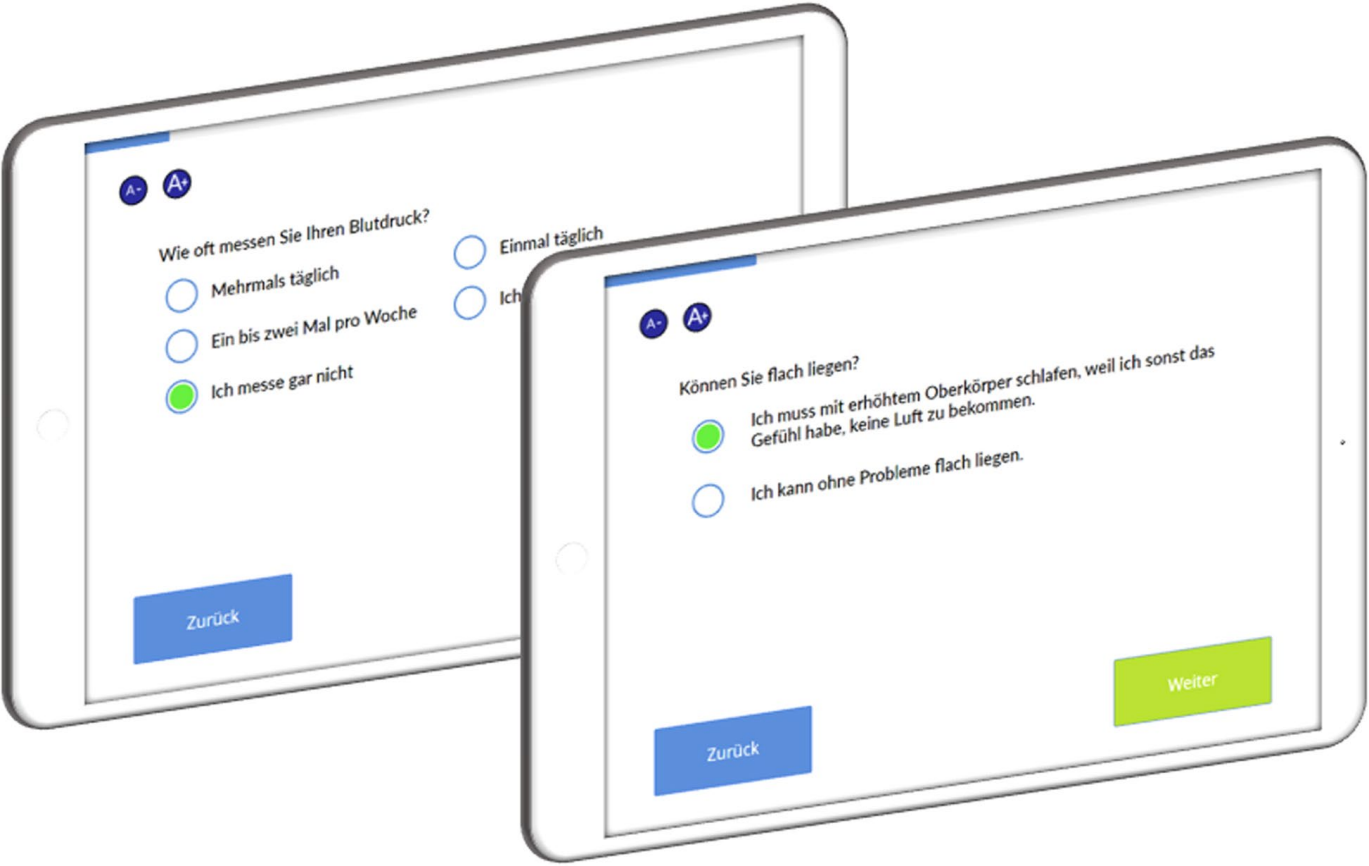
Exemplary visualisation of patient interviews using the INTERVIEW system. The interface is clearly laid out, with buttons that allow the user to change the font size as well as Next and Back below to the questions and answers

The INTERVIEW system is structured as a loosely coupled microservice architecture. While the architecture relies on a number of core services (database, backend, output interface), it can be enhanced easily with additional services to suit the required capabilities for a deployment (e.g., text generator, GDT interface, printer interface, handheld patient record barcode scanner interface, PDF generator etc.). This allows for maximum handling flexibility. The web configuration frontend facilitates the log-in to the backend, the tablet-based patient interview, administration and user management, and the retrieval of anonymised research data. Both the configuration and questionnaire frontends support the visualisation on a desktop computer, tablet, or smartphone, and can be displayed on both an app and a website. For the current project, the frontend was only used on iPads as an app. Figure 3 shows an exemplary illustration of a tablet with the INTERVIEW system interface. The backend implements the content-related processes, such as question selection during the survey or text generation.

#### Content creation and data curation

Technically, the questions and text generation rules are based on a knowledge base that was set up with the cooperating physician in an iterative process, assisted by an IT specialist with knowledge of the syntax. The knowledge base was created in a spreadsheet program as a csv file. The initial content of the interview was based on a paper questionnaire and existing text modules of the physician. Items and the sequence of questions were generated according to the physician’s approach to history taking and mapped accordingly. The finally applied questions and answers are listed in the supplementary files. All of the data and software was stored on the practice’s servers with access via the practice’s password-protected internal network, the data itself was password-protected separately.

### Regulatory and ethical considerations

#### Legal aspects

According to the international guideline of the Medical Device Coordination Group (MDCG 2019–11), software applied in a medical context may be subject to medical device regulation, if it has a “medical purpose on its own” [15]. At the same time, the intended use of a software is of central importance. The developers designed the INTERVIEW system as a generic documentation solution, i.e. not limited to medical applications. For instance, it has been used in schools to survey pupils/teachers and enhance user-friendly documentation. The device regulation further specifies that ‘altering the representation of data for embellishment/cosmetic or compatibility purposes does not readily qualify the software as medical device software’ [15]. Further, purposes such as own reasoning or clinical decision algorithms are not part of the INTERVIEW solution. As described in section “Content creation”, the software was customised to the specific approach to history taking of the cooperating physician, considering the sequence and mode of asking specific questions to elicit specific answers. Therefore, with respect to these definitions and the intended use described above, the INTERVIEW system is not subject to regulation under the medical device directive.

#### Data safety

For reasons of data protection, the INTERVIEW system was deployed locally. The INTERVIEW server and the PMS server were installed within the same environment. Communication between server and tablet was realised in an encrypted and password protected Wi-Fi environment. Patient information was temporarily stored on the INTERVIEW server and deleted after being transferred to the PMS. The INTERVIEW system implements role-based access controls (RBAC) with different account types for technical administrators, anamnesis end devices, assistant staff, and physicians. Depending on the role, different control and data retrieval options are available to users in a web interface in the local network. Assistant staff is only allowed to start and end an interview, while physicians are able to additionally retrieve aggregated data and view quality and feedback trends. During the interview, the data is temporarily cached in the app on the end device, and after completion of the survey and the successful transmission to the INTERVIEW server, all patient data is purged from the device. This excludes potential abuse by subsequent patients handling the same device. Additionally, the data is deleted from the cache when the app is restarted or closed. The collected data itself was stored in the backend and could be accessed via a dashboard in the internal network of the practice. Here, the developers were able to call up the research data as a csv file via a button.

#### Ethical aspects

The implementation of the INTERVIEW system meant its application to all patients in whom the history had to be taken and marked the start of a new routine care process within the cardiology practice. Therefore no informed consent was obtained. Anonymised data retrieval to obtain the here reported aggregated variables was regarded as part of quality control measures of the cardiology practice. The study was conducted in accordance with the Declaration of Helsinki. The Ethics Committee of the responsible State Medical Association (Baden-Wuerttemberg) was consulted and confirmed that formal ethical approval and written informed consent of patients was not required for this research project upon using anonymized data as the physical or psychological integrity of people is not interfered with in reference to §15 of the model professional code of the German Medical Association for physicians in Germany [16].

### Data analysis

Analysis was done using the open-source statistical software R and MS Excel as descriptive workup. As this pilot project is exploratory by nature, statistical testing was kept to a minimum.

## Results

A total of 2,415 completed interviews were registered by the system in the period under consideration. Depending on their individual answers, between 25 and 48 questions were presented in an interview. Relative to the days of use, 12.2 complete cases per day were processed by the system. For details on the characteristics of patients completing the interviews see Table 1.

**Table 1:** Characteristics of participating patients. Age, height, and weight data were corrected for implausible values prior to analysis in order to not distort the results due to typing errors

**Characteristic**	Missing	**All participants** *N* = 2,415
**Age (years)**	1.5%	63.8 (14.7) [18; 101]
**Sex, n (%)**	0%	
*Male*		1,163 (48%)
*Female*		1,252 (52%)
**Height (cm)**	4.0%	169.9 (9.4) [123; 201]
**Weight (kg)**	1.2%	81.9 (19.7) [40; 178]

Data are mean (SD) [min; max], unless indicated otherwise

The technical generation of a text for the physician´s letter was successful in 99.5% of cases. Enclosed is a translated example of a medical history report of the INTERVIEW system^2^:

*“Mr. XX presents for cardiological control. He reports constant exercise tolerance with dyspnea even during easy physical exertion (corresponding to NYHA III). Blood pressure values measured at home (around 150/90 mmHg, HR 82/min) consistently show circulatory stability. Complaints in the sense of typical angina pectoris are not reported. In the vegetative medical history good appetite with weight gain of about 1–3 kg within a few months (unwanted) (123 kg, 182 cm, BMI 37 kg/m*^*2*^*). Daily drinking quantity 1.0 L, with no more than one episode of nocturia. Alcohol consumption: less than 1 d/w. Nicotine use up to 30 cigarettes daily for 36 years (54 PY). Peripheral oedema is only present in the evening. Orthopnea and nocturnal respiratory episodes are observed. No recent fever or other signs of infection.”*

### Appropriateness

For technical reasons, the documentation of time stamps informing on start and end of interview was not available right from the start (but from 11 Feb 2021 onwards). Therefore, only 1127 time stamp records were included in this analysis. On average, a patient needed 9:39 min to complete the entire questionnaire (median 8:51 min, SD 3:58 min). On average, 29.5 questions were answered (median 29, SD 2.97). Thus, answering a question required 18.4 (SD 7.6) seconds on average (median 16.9 s, min 3.8 s, max 51.5 s). The maximum completion time was 29:44 min. Also extreme values for completion time were within a time span of 30 min. The completion time thus was judged “appropriate” for practical use.

### Patient acceptance

**Fig. 4:**
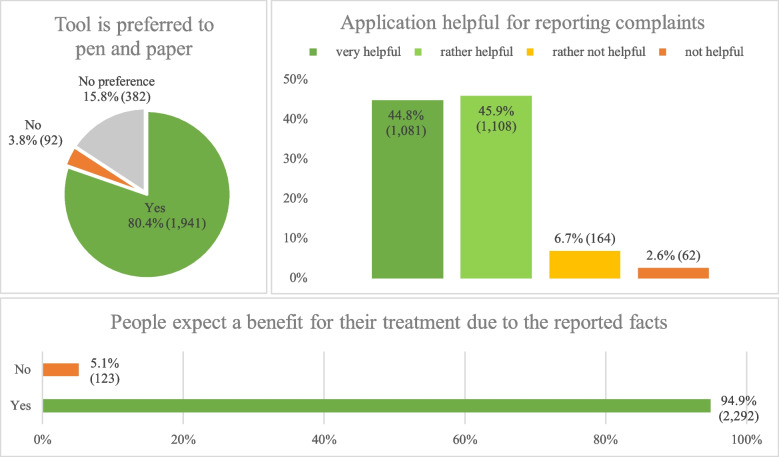
Visualisation of the results of the three questions on the domain of patient acceptance consisting of the questions ‘Do you find a tablet survey more convenient than a paper survey?’ (top left section), ‘How helpful do you feel the questions asked are in terms of assessing your complaints?’ (top right section) and ‘Do you think the survey prior to the physician visit has an advantage for my treatment?’ (bottom section). The three questions were answered by 2,415 patients. Proportion in % and absolute number of patients in () are shown

Figure 4 summarises the patient feedback. The question of whether the tablet survey was more convenient than a paper survey was answered positively by 80%. An additional 16% of respondents had no preference regarding either method. Thus, the tablet questionnaire offered equal or better than status quo experiences for 96% of the patients. The comparison of different age groups showed similar preferences with regard to the interview mode (Table 2). There was a nominally lower preference for the tablet amongst older patients (> 80 years), see Table 2. The question of whether the tablet survey was helpful for the assessment of complaints (pooled consideration of “very helpful” and “rather helpful”) received a very high approval rating of over 90%. The question whether patients expected a benefit for the treatment by using the INTERVIEW system was approved by 95%.

**Table 2:** Distribution of acceptance questions by age group

Characteristic	Total sample	Age group (years)			
	***N* = 2,379**	**18–40 years** ***N* = 177**	**41–65** ***N* = 1,038**	**66–80** ***N* = 854**	**81–101** ***N* = 310**
**Interview preferred to pen and paper**					
*Yes*	1,911 (80%)	151 (85%)	842 (81%)	687 (80%)	231 (75%)
*No*	89 (3,7%)	4 (2,3%)	41 (3,9%)	29 (3,4%)	15 (4,8%)
*No preference*	379 (16%)	22 (12%)	155 (15%)	138 (16%)	64 (21%)
**Tablet survey helpful for reporting complaints**					
*Very helpful*	1,058 (44%)	50 (28%)	434 (42%)	442 (52%)	132 (43%)
*Rather helpful*	1,097 (46%)	110 (62%)	482 (46%)	350 (41%)	155 (50%)
*Rather not helpful*	163 (6,9%)	14 (7,9%)	91 (8,8%)	42 (4,9%)	16 (5,2%)
* Not helpful*	61 (2,6%)	3 (1,7%)	31 (3,0%)	20 (2,3%)	7 (2,3%)
**Interview benefits treatment**					
*Yes*	2,258 (95%)	165 (93%)	977 (94%)	817 (96%)	299 (96%)
*No*	121 (5,1%)	12 (6,8%)	61 (5,9%)	37 (4,3%)	11 (3,5%)

n (%)

A comparative analysis of all age groups revealed only minor variations regarding the appraisal of helpfulness, see Table 2. Digital skepticism was not apparent in older patients. Instead, the age groups 66–80 and 81–101 reported slightly more often that the system was perceived “very helpful” or “rather helpful” (93% each) compared to younger patients in age groups 18–40 years (90%) and 41–65 years (88%), see Table 2. Hence, the INTERVIEW system yielded good patient acceptance according to the a priori definition of the current, i.e. an average of over 80% favourable answers in the three questions.

### Usefulness

In the first interview round, the physician completed 77 questionnaires within 2 weeks. In 40 of the 77 questionnaires (52%), she reported errors in the computer-generated texts. In the plausibility assessment, she graded 32 texts with school grade 1 (“very good”) and 29 texts with grade 2 (good”). Eight times she gave the grade 5 (“not satisfactory”) and four times grade 6 (“insufficient”). When the time saved was inquired, the physician answered all 77 questionnaires with “significantly faster”. The physician also provided 51 free-text responses that were aimed to improve the system: e.g., she noted 22 deficits in items on the cardiovascular situation, and 10 incorrect responses on pectanginal complaints. All feedback forms that had received a grade 5 or 6 for plausibility had one of these two free text items. The feedback form and an aggregated presentation of the incorrect items can be found in the Supplementary Material. Built on this experience, the tool was optimised.

During the second survey, 46 questionnaires were received within a period of 2 weeks. In 70% of the questionnaires, the physician still reported errors in the computer generated text proposals, yet. The physician stated that the use of the INTERVIEW system had made her work “significantly faster”. Particularly noteworthy in terms of frequency were the free-text responses to cardiovascular items: errors were reported in 46% of the responses. Accordingly, the INTERVIEW system was adapted again to improve computer generated outputs.

At the end of the project, the physician was asked again about her experiences. She then reported hardly any errors in the texts. Occasionally there had been some input faults, e.g. if a patient had entered 1000L as the quantity of drink. However, she was able to detect these errors quickly. Overall, the plausibility of the content was good. According to the physician’s perception, using the INTERVIEW system had accelerated workflows saving her about one hour per day in documentation time. She added that the anamnesis was usually sufficiently comprehensive for her needs and that she only had to make small changes, if any. Thus, overall, the system can be regarded “useful” according to the definition set a priori for the current project.

### Organisational and technical findings

The system was used by the practice with similar frequency over the months. In the interval, there were temporary outages in system use due to technical difficulties within the practice, which, however, were of minor importance. The local implementation of the system and the adaptation to the practice environment required a considerable amount of time for the developers. Since there was initially no VPN connection available in the practice, the developers had to provide on-site support, which required considerable effort. During the implementation period, content development was coordinated between the participating physician and the developers. Further, mapping the medical content to the questionnaire logic language was not trivial at times. Expert support and access by the developers to the locally installed system was relevant, although less frequently needed once initial technical difficulties had been resolved. By the end of the period under consideration the practice was allowed to continue to use the INTERVIEW system and receives support to the extent possible from the developers on a voluntary basis.

## Discussion

The here reported INTERVIEW system was tested in the clinical setting of a resident cardiologist, in Germany. The three domains under review, i.e. appropriateness, patient acceptance and usefulness, all received a positive evaluation. The INTERVIEW system fitted well into the time constraints and organisational challenges of the practice, was appreciated by patients, and was favourably perceived by the participating physician. As such, the system made a relevant contribution to essential organisational processes as it facilitated the user-friendly recording of self-reported patient anamnesis, its presentation in an appropriate textual format ready for use in the physician letter, and allowed to save time when using the tool. The various aspects and limitations of the project are discussed as follows.

### Appropriateness

Technical tools must fit into the workflows of medical facilities. If tools delay the workflow, they will not or only reluctantly be used. The average time to complete an interview was about 10 min in the current project. This leaves room for extension of content adapted to specialized needs, e.g. as a dedicated questionnaire for university outpatient clinics. However, the current project showed that it is possible to collect a comprehensive cardiological anamnesis under real-life conditions.

### Patient acceptance

The literature lists a multitude of studies according to which patients have a positive attitude towards tablet-based interviewing, consistent with the results present here [13, 17–21]. Older people tend to have less contact with new technologies and therefore, as expected, less preference for their use [22]. Of note, we observed that the preference for being interviewed via tablet was only slightly lower in older compared to younger people (see Table 2), and more often indicated “no preference”. Thus, overall patient acceptance and the quality of the customised system appeared satisfactory, in particular as the interface was judged user-friendly. Younger patients were slightly less likely to state that the interview was "very helpful" with regard to their treatment. Disease and symptom burden increase with age. Items of interest, such as high cholesterol, high blood pressure or nocturia, are less common in young patients. Accordingly, these questions may appear less helpful. As a caveat, the design of the questions for the assessment of patient acceptance may have biased patient response. This aspect is discussed in the limitations section.

### Usefulness

At the beginning of the project, generated texts were judged faulty by the physician with respect to spelling and grammar, whereas they were rated medically plausible. Questions and answers were adapted in the course of the project, and were close to optimal in its final stage. From the physician´s point of view, a significant added value could be the time saving of approximately one hour per day when using the INTERVIEW system. Interestingly, the physician reported major time savings right from the start, even with texts containing errors. In view of the lack of free-text feedback on items of the vegetative anamnesis, the 14 vegetative anamnesis, for example, appear to have been collected appropriately right from the beginning of the implementation. Items such as nocturia, smoking history, weight development or familial disposition were perceived to provide relevant information. To collect this information by the physician requires time that was saved by using the INTERVIEW solution.

### Physician–patient relationship

The advice and attention a patient receives seems to be important for a trusting physician-patient relationship, especially with specialists with whom one has less contact. Critically asked: What does it mean for the physician-patient relationship when the patient takes the medical history?

It should be noted that for the use of the INTERVIEW system the hitherto established processes were not altered to a large degree. An anamnesis in the format of a personal interview between physician and patient was still taken, but it is expectedly more focused. Consequently, patients frequently stated that they expected a benefit for the treatment by answering the questions prior to seeing the physician. This suggests their confidence in the care process and may thus positively affect the physician-patient relationship. At the same time, the patient is likely to be better prepared for the subsequent consultation, reducing the information distance between physician and patient. Verlinde et al. emphasized that empowering patients for a more effective exchange might improve the physician-patient relationship [23].

Of note, introducing new technology might cause divergent effects in this relationship. As reported be Hedian et al., patients perceived the physician consultation using electronic health record (EHR) as more efficient and were satisfied, but physicians assumed a deterioration in patient satisfaction or negative effects due to reduced eye contact [24]. This is consistent with the positive perception of patients in our project. However, the physician´s view of the tool in this project was also quite positive. Hedian et al. emphasize that deploying an EHR means restructuring documentation and organizational requirements on the side of the physician, and hypothesize that physicians project their own dissatisfaction with digital systems onto the patients and into the physician-patient relationship [24]. The literature shows that the satisfaction of physicians decreases with increasing workload and that patient satisfaction correlates with physician satisfaction [25].

This is a central point when using the INTERVIEW system. In contrast to traditional EHRs and paper questionnaires, the INTERVIEW system can reduce the physician´s documentation workload. This allows the physician to focus on the patient, knowing that the content of interest has already been captured in adequate quality.

Conversely, Hedian et al. also state that there is no evidence that an EHR may have a negative impact on the empathic ability of physicians [24]. It can therefore be expected that the physician is at least equally attentive due to the eased documentation during the consultation. It should be noted, however, that in a resource-constrained health care system, such time savings will be priced in over the medium term, reducing any potential positive effects.

### Organisational and technical findings

The German Federal Ministry of Health (BMG) called forward the development of applications to increase the efficiency of care and administrative processes as part of its digitisation strategy [26]. The knowledge-based technology used in this project was not a large language model generally. Rather, the approach presented allows for transparent, reproducible and unambiguous decision-making. Transparency and comprehensibility are of crucial importance when it comes to medical decisions. However, with regard to the further development of the content of questions or answers, human reasoning will always be necessary for the creation of new or updated medical content. Also from an organisational point of view the applied approach proved to be time-consuming. Hence, the INTERVIEW system meets key requirement of the digitisation strategy of the German Federal Ministry of Health, namely the provision of a service that can also be used by all patients, even those with low digital affinity [26]. Addressing the implementation and maintenance costs also appears to be relevant. Here, providing the software via cloud hosting would be a favourable approach to keep maintenance costs low and the creation of a scalable solution.

### Future perspectives for research and implementation

The INTERVIEW system may be considered a feasible approach that can be recommended for further research and testing. Beyond this pilot implementation, however, a comprehensive financial framework is required to ensure sustainability. A scalable provision of the INTERVIEW system should include a module for research purposes. The INTERVIEW system already has an interface for anonymised retrieval of patient data, which easily can be expanded. With this type of medical history taking, the input and output language can be different, allowing language barriers to be dealt with effectively. Another interesting feature for the INTERVIEW system would be a platform for the creation and exchange between users with regard to the mapping of content-related questions. Here, a questionnaire creation and exchange platform for independent operation of applying physicians would be valuable. A forum feature would allow for reconciliation of items with direct peer review, and direct validation of content through patient trialling at different sites.

### Limitations and strengths

Limitations of this pilot implementation deserve mentioning. The system was set up at one practice with one physician using the tool. With regard to the previously mentioned institution- and personality-specific influencing factors, several sources of bias are conceivable, especially for the usefulness endpoint [11, 12]. Patient acceptance was assessed using non-validated questions, some of them with phrasings that may have introduced bias due to their directive nature (e.g., asking how helpful the system is when reporting complaints). Patient acceptance of the INTERVIEW system may therefore have been overestimated. Carefully structured post-visit interviews could be also used in future evaluations to overcome this. Therefore, the results reported here await confirmatory studies with a more robust design before a broader conclusion can be drawn.

Strengths of the present project are the long-time interval of data collection resulting in a high number of consecutive patients analysed. The practice studied was located in a rural region and the digital affinity among patients was perceived to be rather low, so that limitations in this direction based on the monocentric collective seem less likely.

## Conclusions

The current pilot project in 2,415 consecutive outpatients attending a cardiological residential practice in Germany suggests that the deployment of the INTERVIEW system is feasible. This innovative approach allowed to retrieve the self-reported medical history of a patient prior to physician contact with sufficient precision to support the routine care process of the observed practice. The system was regarded appropriate in routine care, well accepted by patients and useful, i.e. time-saving, from the physician´s perspective. Because bias could not be well quantified in this uncontrolled pilot project, future studies with a more rigorous study design and in alternative settings are needed to confirm and expand these promising findings.

## Supplementary Information

Enclosed we provide an overview of the questions and answers used in the practice. The questions were mainly asked in the order shown; due to the rule-based nature of the INTERVIEW system, individual questions were asked depending on the previous answer. In addition, we provide the questionnaires to the physician as well as on the aggregated feedback.

Supplementary Material 1.

Supplementary Material 2.

Supplementary Material 3.

Supplementary Material 4.

## Data Availability

The datasets analysed during the current project are available from the corresponding author upon reasonable request.

## Footnotes

1 Exceptions were numerical entries; respective data entry fields could remain empty.

2 Translated from German.
