# Supplementary material for: Computer-assisted medical history taking prior to patient consultation in the outpatient care setting: a prospective pilot project
Source: BMC Health Serv Res. 2024 Dec 18;24:1616. doi: 10.1186/s12913-024-12043-3 (PMC11658432; doi:10.1186/s12913-024-12043-3)
Supplement: Supplementary file 1 — Supplementary Material 1. [file 12913_2024_12043_MOESM1_ESM.docx]

| **Item** | **Type (conditional calling)** | **Question and answer** |
| --- | --- | --- |
|  |  |  |
| First presentation | **SC** | **Are you here for the first time or for a follow-up?** |
|  | A | For the first time |
|  | A | For follow-up |
|  |  |  |
| Gender | **SC** | **What is your gender?** |
|  | A | male |
|  | A | female |
|  |  |  |
| Weight | **NUM** | **Please enter your weight (kg)!** |
|  |  |  |
| Body size | **NUM** | **Please enter your size in centimeters!** |
|  |  |  |
| Age | **NUM** | **How old are you?** |
|  |  |  |
| Angina pectoris (1) | **SC** | **Do you repeatedly suffer from pressure pain or stitching pain in your chest?** |
|  | A | Yes |
|  | A | No |
|  |  |  |
| Angina pectoris (2) | **MC** ( *Yes* to previous question) | **What is the context for this pressure or stabbing pain in the chest?** |
|  | A | ALWAYS occurs during physical or mental exertion |
|  | A | Disappears when resting/stress subsides |
|  | A | None of the statements apply |
|  |  |  |
| Palpitations | **SC** | **Have you noticed a ‘racing heart’ lately that makes you feel dizzy?** |
|  | A | Yes |
|  | A | No |
|  |  |  |
| Dizziness | **SC** ( *Yes* to previous question) |  |
|  | A |  |
|  | A |  |
|  |  |  |
| Syncope | **SC** | **Do you find yourself in everyday situations in your life where you feel so dizzy that you threaten to faint?** |
|  | A | Yes |
|  | A | No |
|  |  |  |
| Leg edema | **SC** | **Do you notice swollen ankles or lower legs every day?** |
|  | A | Only in the evening |
|  | A | Permanently |
|  | A | No |
|  |  |  |
| Compression stockings | **SC** (Not *No* to previous question) | **Do you wear compression stockings for those swollen ankles or lower legs?** |
|  | **A** | Yes |
|  | **A** | No |
|  |  |  |
| Apnea symptoms | **SC** | **Are you aware of nocturnal breathing interruptions?** |
|  | A | Yes |
|  | A | No |
|  |  |  |
| Orthopnea | **SC** | **Do you always get enough air when you lie flat on your back?** |
|  | A | Yes |
|  | A | No |
|  |  |  |
| infection | **SC** | **Have you recently had a serious infection, fever or cough?** |
|  | A | Yes, still present |
|  | A | Yes, but not anymore |
|  | A | No |
|  |  |  |
| Resilience | **SC** | **How has your physical resilience with regard to shortness of breath developed?** |
|  | A | Remained the same |
|  | A | Worsened |
|  | A | Got better |
|  | A | I don't notice any shortness of breath |
|  |  |  |
| NYHA | **SC** | **Please check the box with the description that most closely matches your physical condition. If in doubt, please choose according to the worse condition.** |
|  | A | I can perform all physical activities without becoming short of breath. |
|  | A | I get short of breath during more strenuous activities (e.g. walking uphill, climbing more than 2 flights of stairs). |
|  | A | I get short of breath during everyday activities (e.g. walking on level ground, climbing more than 1 flight of stairs). |
|  | A | I have shortness of breath at rest and am mainly confined to my home. When I'm physically active, I become short of breath, tired, or experience palpitations. |
|  |  |  |
| Appetite | **SC** | **How has your appetite been lately?** |
|  | A | I have no appetite. |
|  | A | I have little appetite |
|  | A | My appetite is good |
|  |  |  |
| Weight development | **SC** | **How has your weight changed in the last month?** |
|  | A | Remained the same |
|  | A | Gained weight intentionally |
|  | A | Gained weight unintentionally |
|  | A | Lost weight intentionally |
|  | A | Lost weight unintentionally |
|  |  |  |
|  |  |  |
| Weight difference (1) | **NUM** (if asked about weight development, indicate *weight gain* ) | **How much weight have you been gaining? (kg)** |
|  | A | Approx. 1-3 kg |
|  | A | Approx. 3-6 kg |
|  | A | More than 6 kg |
|  |  |  |
| Weight change (2) | **NUM** (if asked about weight development, indicate *weight loss* ) | **How much weight have you been losing? (kg)** |
|  | A | Approx. 1-3 kg |
|  | A | Approx. 3-6 kg |
|  | A | More than 6 kg |
|  |  |  |
| Weight Period | **NUM** (If you are asked about weight development, please indicate a *change* ) | **Within what period of time did this change in weight take place? Please select the most appropriate answer** |
|  | A | Within a few weeks |
|  | A | Within a few months |
|  | A | For more than half a year |
|  |  |  |
| Drinking amount | **NUM** | **How many liters of liquids do you drink daily (including tea/coffee)?** |
|  |  |  |
| Alcohol | **SC** | **How many alcoholic drinks do you consume per week? (1 alcoholic drink corresponds to 0.33 L beer or 0.2 L wine or 0.01 L liquor)** |
|  | A | I don't drink any alcohol |
|  | A | Less than one per week |
|  | A | One to two per week |
|  | A | Three to five per week |
|  | A | More than five per week |
|  |  |  |
| Nocturia | **SC** | **How often do you have to go to the toilet at night?** |
|  | A | 0 - 1 times |
|  | A | 2 – 4 times |
|  | A | More than 4 times |
|  |  |  |
| Nicotine abuse (1) | **SC** | **Do you smoke?** |
|  | A | Yes |
|  | A | No |
|  | A | No, but I used to smoke |
|  |  |  |
| Nicotine abuse (2) | **SC** ( *Yes* or *Previously* on Smoking Question) | **How many cigarettes do/did you smoke per day?** |
|  | A | 1 to 5 cigarettes per day |
|  | A | Up to 10 |
|  | A | Up to 20 |
|  | A | Up to 30 |
|  | A | Up to 40 |
|  | A | Over 40 |
|  |  |  |
| Nicotine abuse (3) | **NUM** ( *Yes* or *previously* on smoking question) | **Approximately how many cigarettes do/did you smoke per day?** |
|  |  |  |
| Nicotine abuse (4) | **NUM** ( *Yes* or *previously* on smoking question) | **How old were you when you started smoking?** |
|  |  |  |
| Nicotine abuse (5) | **NUM** ( *previously* on smoking issue) | **How old were you when you quit smoking?** |
|  |  |  |
| Blood pressure measurement | **MC** | **What applies to you?** |
|  | A | I measure my blood pressure myself. |
|  | A | My family doctor measures my blood pressure. |
|  | A | I don't know my blood pressure values. |
|  |  |  |
| Doctor Feedback | **SC** (selection of *family doctor* after previous blood pressure question) | **What does your doctor say about your blood pressure?** |
|  | A | Too high |
|  | A | All right |
|  | A | Too low |
|  | A | I do not know |
|  |  |  |
| Systole | **NUM** (Selection *even* with previous blood pressure question) | **Please enter the average measured upper blood pressure value, e.g. 120.  If you do not know the value, please enter 0!** |
| Diastole | **NUM** (Selection *even* with previous blood pressure question) | **Please enter the average lower blood pressure value measured, e.g. 90. If you do not know the value, please enter 0!** |
|  |  |  |
| Heart rate (1) | **SC** | **Do you know your average heart rate?** |
|  | A | Yes |
|  | A | No |
|  | A | I am not sure |
|  |  |  |
| Heart rate (2) | **NUM** (Selection of *Self* for previous blood pressure question AND selection of *Yes* for previous pulse question)) | **Please enter your average pulse, e.g. 70.  If you don't know the value, please enter 0!** |
|  |  |  |
| Allergies (1) | **SC** | **Do you have any allergies?** |
|  | A | Yes |
|  | A | No |
|  |  |  |
| Allergies (2) | **MC** (select *yes* if previous allergy question) | **What allergies are known?** |
|  | A | Contrast agent |
|  | A | Antibiotics |
|  | A | Food |
|  | A | Plaster/Dressing |
|  | A | None of the above |
|  | A | I am not aware of any |
|  |  |  |
| Anaphylaxis | **SC** | **Have you ever had a life-threatening allergic reaction?** |
|  | A | Yes |
|  | A | No |
|  |  |  |
| CVRF | **MC** | **Which of the following pre-existing conditions applies to you?** |
|  | A | Elevated cholesterol levels |
|  | A | Diabetes |
|  | A | High blood pressure |
|  | A | None of the above |
|  |  |  |
| Familial disposition (1) | **SC** (From Phase III onwards, the item on family disposition was divided into several individual questions in order to reduce complexity. The following questions were conditioned accordingly depending on previous answers. Not explained in detail for the sake of clarity.) | **Have your parents, siblings or children had a heart attack or stroke?** |
|  | A | Yes |
|  | A | No |
|  |  |  |
| Familial disposition (2) | **SC** (due to screening question on familial disposition) | **Which of the named relatives had a heart attack or stroke?** |
|  | A | Mother |
|  | A | Father |
|  | A | Sister |
|  | A | Brother |
|  | A | Daughter |
|  | A | Son |
|  |  |  |
| Familial disposition (3) | **SC** (due to screening question on familial disposition) | **Was your mother younger than 65 years of age when she had the heart attack/stroke?** |
|  | A | Yes |
|  | A | No |
|  |  |  |
| Familial disposition (4) | **SC** (due to screening question on familial disposition) | **Was your sister younger than 65 years of age when she had the heart attack/stroke?** |
|  | A | Yes |
|  | A | No |
|  |  |  |
| Familial disposition (5) | **SC** (due to screening question on familial disposition) | **Was your daughter younger than 65 years of age when she suffered the heart attack/stroke?** |
|  | A | Yes |
|  | A | No |
|  |  |  |
| Familial disposition (6) | **SC** (due to screening question on familial disposition) | **Was your father younger than 55 years of age when he suffered the heart attack/stroke?** |
|  | A | Yes |
|  | A | No |
|  |  |  |
| Familial disposition (7) | **SC** (due to screening question on familial disposition) | **Was your brother younger than 55 years of age when he had the heart attack/stroke?** |
|  | A | Yes |
|  | A | No |
|  |  |  |
| Familial disposition (8) | **SC** (due to screening question on familial disposition) | **Was your son younger than 55 years of age when he suffered the heart attack/stroke?** |
|  | A | Yes |
|  | A | No |
|  |  |  |
| Opinion - Helpful | **SC** | **How helpful do you feel the questions asked are in terms of assessing your complaints?** |
|  | A | Very helpful |
|  | A | Rather helpful |
|  | A | Rather not helpful |
|  | A | Not helpful |
|  |  |  |
| Opinion - Pleasant | **SC** | **Do you find a tablet survey more convenient than a paper survey?** |
|  | A | Yes |
|  | A | No |
|  | A | I do not care |
|  | A |  |
| Opinion - Advantage | **SC** | **Do you think the survey before the doctor visit has an advantage for the treatment?** |
|  | A | Yes |
|  | A | No |
|  |  |  |
|  |  |  |

Legend: SC (SingleChoice): only one answer can be selected; MC (MultipleChoice): several answer options can be selected; NUM (numerical question): enter a number using the digital numeric keypad of the tablet.

A (Answer): Answer option for the item above.
