# Supplementary material for: Computer-assisted medical history taking prior to patient consultation in the outpatient care setting: a prospective pilot project
Source: BMC Health Serv Res. 2024 Dec 18;24:1616. doi: 10.1186/s12913-024-12043-3 (PMC11658432; doi:10.1186/s12913-024-12043-3)
Supplement: Supplementary file 2 — Supplementary Material 2. [file 12913_2024_12043_MOESM2_ESM.docx]

**Evaluation Sheet: Physisican Feedback (English translation from German language)**

Responses obtained in period (I), from 31.8.2020–28.9.2020: n=77

Responses obtained in period (II), from 28.9.2020–13.10.2020, n=46

**Does the proposed text contain errors?** (Yes/No)

**I II**

**Yes:** 40 32

**No:** 37 14

**No input:** 1 -

**Is the proposed text plausible (correct in terms of content)?** (Grade 1-6)

**I II**

**Graded with 1:** 32 13

**Graded with 2:** 29 4

**Graded with 3:** 2 3

**Graded with 4:** 1 6

**Graded with 5:** 8 -

**Graded with 6:** 4 1

**No input:** 2 18

**My perception was, when using the documentation tool, I was .......... than without the tool!** (“significantly slower”, “slower”, “similarly fast”, “faster”, “significantly faster”)

**I II**

**Significantly faster:** 77 45

**No input:** - 1

**Comments:**

Under the comments section, there was keyword feedback, which is listed below in clusters.

**I II**

**Helpful/good/o.k./super/‘**✓**’:** 9 11

**For the item NYHA:**  2 1

**NYHA II:** 1 2

**NYHA III:** 1 -

**NYHA IV:** 1 -

**Cardiovascular situation:** 22 21

**Palpitations:** 4 3

**(Former) tobacco consumption:** 2 2

**Symptoms of angina pectoris:** 10 12

**Cardiovascular (risk) factors:** 3 1

**Hypertension:**  3 -

**Obesity:**  - 1

**Vertigo:**  2 -

**Syncope:** 2 -

**Family medical history:**  2 -

**Body weight:** 1 -

**Alcohol:** 1 -

**Leg oedema:** 1 -

**Infection:** 1 -

**No input:** 26 2
