# Supplementary material for: Computer-assisted medical history taking prior to patient consultation in the outpatient care setting: a prospective pilot project
Source: BMC Health Serv Res. 2024 Dec 18;24:1616. doi: 10.1186/s12913-024-12043-3 (PMC11658432; doi:10.1186/s12913-024-12043-3)
Supplement: Supplementary file 3 — Supplementary Material 3. [file 12913_2024_12043_MOESM3_ESM.docx]

**Evaluation form (English translation from German language)**

Does the text proposal contain errors?

**Yes**

**No**

Evaluation in school grades

Is the proposed text plausible (correct in terms of content)?

**1**

**2**

**3**

**4**

**5**

**6**

Complete the sentence

My perception was, when using the documentation tool, I
was _________ than without the tool.

significantly slower

slower

significantly faster

faster

similarly fast

Comments:
