# Supplementary material for: Computer-assisted medical history taking prior to patient consultation in the outpatient care setting: a prospective pilot project
Source: BMC Health Serv Res. 2024 Dec 18;24:1616. doi: 10.1186/s12913-024-12043-3 (PMC11658432; doi:10.1186/s12913-024-12043-3)
Supplement: Supplementary file 4 — Supplementary Material 4. [file 12913_2024_12043_MOESM4_ESM.docx]

Interviewee (name): Date of interview:

Thank you for taking the time to provide feedback again on the use of the INTERVIEW system in your practice. The questions essentially correspond to those on the evaluation forms that you filled out when writing the patient reports to the GP. Now that the system is running stable, we again are interested to learn how you perceived working with the tool. If the question is not clear, please feel free to ask. You are also welcome to tell me about experiences that go beyond the question. I would make a note of your answers during the interview.

Did the text items proposed by the tool that you edited – in a period of about the last two weeks – contain errors?

Were the proposed text items plausible and – as far as can be assessed – correct in terms of content?

How has the use of documentation support affected your work? Please complete the sentence:

My perception was, when using the documentation tool, I was _________ than without the tool. (Significantly faster, faster, similarly fast, slower, significantly slower).
